# Flapping Tail Membrane in Bats Produces Potentially Important Thrust during Horizontal Takeoffs and Very Slow Flight

**DOI:** 10.1371/journal.pone.0032074

**Published:** 2012-02-29

**Authors:** Rick A. Adams, Emily R. Snode, Jason B. Shaw

**Affiliations:** School of Biological Sciences, University of Northern Colorado, Greeley, Colorado, United States of America; University of Western Ontario, Canada

## Abstract

Historically, studies concerning bat flight have focused primarily on the wings. By analyzing high-speed video taken on 48 individuals of five species of vespertilionid bats, we show that the capacity to flap the tail-membrane (uropatagium) in order to generate thrust and lift during takeoffs and minimal-speed flight (<1 m ^s−1^) was largely underestimated. Indeed, bats flapped the tail-membrane by extensive dorso-ventral fanning motions covering as much as 135 degrees of arc consistent with thrust generation by air displacement. The degree of dorsal extension of the tail-membrane, and thus the potential amount of thrust generated during platform launches, was significantly correlated with body mass (P = 0.02). Adduction of the hind limbs during upstrokes collapsed the tail-membrane thereby reducing its surface area and minimizing negative lift forces. Abduction of the hind limbs during the downstroke fully expanded the tail-membrane as it was swept ventrally. The flapping kinematics of the tail-membrane is thus consistent with expectations for an airfoil. Timing offsets between the wings and tail-membrane during downstrokes was as much as 50%, suggesting that the tail-membrane was providing thrust and perhaps lift when the wings were retracting through the upstoke phase of the wing-beat cycle. The extent to which the tail-membrane was used during takeoffs differed significantly among four vespertilionid species (P = 0.01) and aligned with predictions derived from bat ecomorphology. The extensive fanning motion of the tail membrane by vespertilionid bats has not been reported for other flying vertebrates.

## Introduction

The evolution of flight in mammals occurred more than 50 million years ago and led to today's diversity of bats that compose nearly 24% of all living mammalian species [1]. The dawn of bat flight was evolutionarily independent from events that gave rise to birds and pterosaurs, the only other groups of vertebrates known to have evolved flight. Accordingly, the form and function of bat wings differ considerably from birds and pterosaurs and the unique nature of bat flight has stimulated much interest in habitat use and foraging ecology of various species [2]. Although studies of flight using wind tunnels have been pervasive, increasingly research has focused on free-flight kinematics in bats which may deliver a more realistic interpretation of how bats use their wings in nature [3]–[10].

Undoubtedly, the nearly singular emphasis on the wings (hand- + arm-wings) of bats as the major adaptive character for flight is warranted and expected. However, such focused research has somewhat diverted investigations of other potentially important attributes and dimensions of flight. For example, an aspect of bat flight not adequately investigated is the use of the tail-membrane (uropatagium) as a contributor to flight performance. Although some insectivorous bats are known for using their tail-membrane to catch insects in flight and also in some elements of flight control [11]–[12], the extent to which bats invest their tail-membrane to augment flight performance has rarely been considered. Two investigators [10], [13], [14] have speculated that the tail-membrane may produce lift during flight and one [10] has noted slight upward and downward motions of the tail-membrane in synchrony with the wingbeat cycle during wind tunnel flights in the brown long-eared bat (*Plecotus auritus*, Vespertilionidae).

Herein we provide the first evidence that vespertilionid bats are capable of extensively flapping their tail-membrane in coordination with the wings and offer a testable model wherein the mechanics and kinematics of the tail-membrane are consistent with expectations for the production of thrust and lift. We hypothesize that the tail-membrane in vespertilionid bats has a significant role in providing thrust and lift during takeoff events at very low flight speeds (<1 m ^s−1^) particularly at moments when the wings offer minimal contributions to flight. We predict that the tail-membrane motions will be consistent with expectations surrounding maximizing positive force production and minimizing production of negative lift forces. We also predict that the degree (extent) of tail-membrane flapping will be correlated with the body mass of individuals of similar wing dimensions because greater thrust is required to lift greater mass from a stationary position. We tested our hypothesis and predictions by filming individuals from five species of vespertilionid bats launching from a horizontal platform expected induce significant flight stress during takeoff.

## Results

### Bats captured and filmed

We filmed 95 individuals of five species of vespertilionid bats as they launched from a horizontal platform after being held stationary by hand. From these, 48 videos met the criteria for analysis (minimal pitch, roll, and yaw during takeoff, ability to track wing tip, wrist, and tail-membrane tip motions throughout beat cycles, as well as smooth, unfettered takeoffs from start-points). Numbers of videos used per bat species were: 12 long-eared myotis, *M. evotis*; 14 little brown myotis, *M. lucifugus*; 7 fringed myotis, *M. thysanodes*; 4 Townsend's big-eared bats, *Corynorhinus townsendii*, and 11 pallid bats, *Antrozous pallidus*. All myotis species and Townsend's big-eared bats were wild-caught and filmed in the field. Pallid bats were filmed at the University of Wyoming, Laramie, USA, where a free-flying, captive colony is held.

### Takeoff sequencing

**Figure 1:**
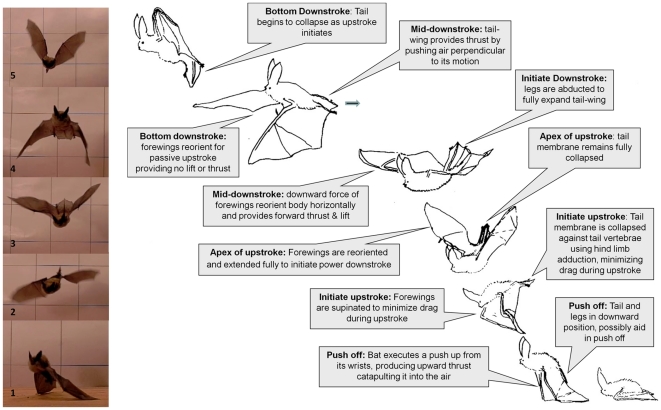
Orchestrating a tail-membrane flap. **A**) 1–2. Fringed myotis (*Myotis thysanodes*) illustrating adduction of its rear limbs to collapse the tail-membrane during the upstroke. 3–4. Abduction of the hind-limbs at the top of the upstroke occurs in preparation for the tail-membrane downstroke, thereby maximizing surface area and air displacement leading to rearward thrust. 5. Collapsing of the tail-membrane at the bottom of the downstroke in preparation for the next upstroke. B) Sequence drawings illustrating motion and timing between wings and tail-membrane motions during initial phase of a platform takeoff by Townsend's big-eared bat (*Corynorhinus townsendii*).

The push-off kinematics and mechanics of horizontal takeoffs have been described for the ground-dwelling common vampire bat (Phylostomidae: *Desmodus rotundus*) and, although push-off mechanics was not described in a study of the dog-faced fruit bat (Pteropodidae: *Cynopterus brachyotis*), calculation of power production by the wings during horizontal takeoffs were made [15], [16]. For vespertilionid bats used in our study, horizontal takeoffs involved a similar push-off to that described for *D. rotundus*, wherein the wrist and thumb acted as the primary launch points. However, whereas *D. rotundus* launched with a head-up body posture, vespertilionid bats typically rocked forward onto their wrists, resulting in a head-down posture with the body-axis tilted at times 30–45 degrees below the horizontal (Figure 1A). Subsequent rotation of the body to a horizontal plane occurred quickly within the first wing and tail-membrane beat cycles. In some cases, however, myotis bats did launch with a head-up posture similar to *D. rotundus*, but this was relatively rare (*n* = 3). Curiously, the pallid bat (*Antrozous pallidus*), a ground foraging species, did typically launch itself with a head-up posture similar to *D. rotundus*, or used a level body-axis orientation. Townsend's big-eared bat (*C. townsendii*, *N* = 4) used a very steep ascent during takeoff nearing 60° to the horizontal before leveling off in flight (Figure 1B), whereas myotis species typically showed a less dramatic ascent angle (∼30°). The pallid bat (*A. pallidus*, *N* = 11) also at times used a very steep angle of ascent with one individual launching at an angle of 69°.

### Complex orchestration of tail-membrane flapping mechanics

By filming bats head-on as they launched from the horizontal platform, we revealed that the tail-membrane complex (hind limbs, tail, and tail-membrane) undergoes intricate and coordinated motions, as do the wings, to minimize downward forces during the upstroke and maximize air-displacement throughout the downstroke (Figure 1A, Videos S1 and S2). By adducting the rear appendages during the upstroke, individuals fully collapsed the tail-membrane, thereby minimizing its area and downward air pressure across the surface (Figure 1A, panels 1 and 2). When the tail-membrane reached the top of the upstroke (Figure 1A, panel 2), the legs were abducted to fully extend the tail-membrane (Figures 1A, panel 3) as it was swept through the downstroke. Fanning motion of the tail-membrane (Figure 1A, panels 3 and 4; Figure 1B, Video S3) would displace air perpendicular to the sweeping motion thereby generating thrust in a rearward direction [17]. The first tail-membrane cycle ended with adduction of the legs at the bottom of the downstroke in preparation for the next upstroke (Fig. 1A, panel 5).

Another important component of the tail-membrane in flight was the ability to alter stroke timing (exemplified in Figure 1A, panel 4), wherein the timing of the tail-membrane downstroke lagged behind the downstroke of the wings by nearly 50%. Lagging the downstroke of the tail-membrane behind that of the wings may serve to provide timely lift and thrust during takeoffs at very slow flight speeds (<1 m ^s−1^) [18], [19]. Although the formation of closed-loop ring vortices have been shown to contribute to lift during the upstroke in wind tunnel flight speeds between 5.0±0.1 m ^s−1^and 6.7±0.4 m ^s−1^ in *C. brachyotis* [20], tests done on the greater horseshoe bat (Rhinolophidae: *Rhinolophus ferrumequinum*) and the long-eared bat (Vespertilionidae: *Plecotus auritus*) flying at speeds below 3 m ^s−1^, showed ‘little to no useful lift’ produced by the wings during the upstroke phase [18], [19]. In addition, at speeds below 3 m ^s−1^ in the nectar-feeding bat (Phyllostomidae: *Glossophaga soricina*), unstable air flow dynamics result in unfavorable (negative) thrust and lift production during the upstroke. However, there was some indication that at the top of the upstroke, inverting the camber of the hand-wing tips by supination potentially produced some thrust [19]. Integrating our data for vespertilionid bats, extensive investment of fanning motions of the tail-membrane during very slow flight from horizontal launch to cruising speed indicates supportive thrust and/or lift generation.

### Degrees of synchrony between wings and tail-membrane motions

Temporal offset patterns, particularly between downstroke timings of the wings and the tail-membrane were quite typical among the species tested. Upon takeoff, both wings moved through the upstroke in tandem to their top positions. However, there was clear variation of synchronized motions between the wings and tail-membrane during initial downstrokes among individuals and the degree of synchrony varied even across wing cycles within the same launch event. Commonly, but not always, the first downstrokes initiated an offset timing (we term these ‘oscillation offsets’) between wings and the tail-membrane. In most cases, the opening downstroke asynchrony was driven by a time-lag in downward motion of the tail-membrane relative to the downward motion of the wings leading to variation in cycling times. For the little brown bat (*M. lucifugus*, N = 14) the mean wing and tail-membrane overall cycle times (top of upstroke through bottom of downstroke to top of upstroke) were not significantly different (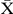
_wing_ = 0.07 seconds, 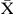
_tail-membrane_ = 0.069 seconds; T-value = 1.33, P = 0.89). However, standard deviations of the tail-membrane cycles were twice those measured for the wings (SD_wings_ = 0.0079, SD_tail-membrane_ = 0.017) indicating greater relative variability in spatio-temporal use of the tail-membrane.

**Figure 2:**
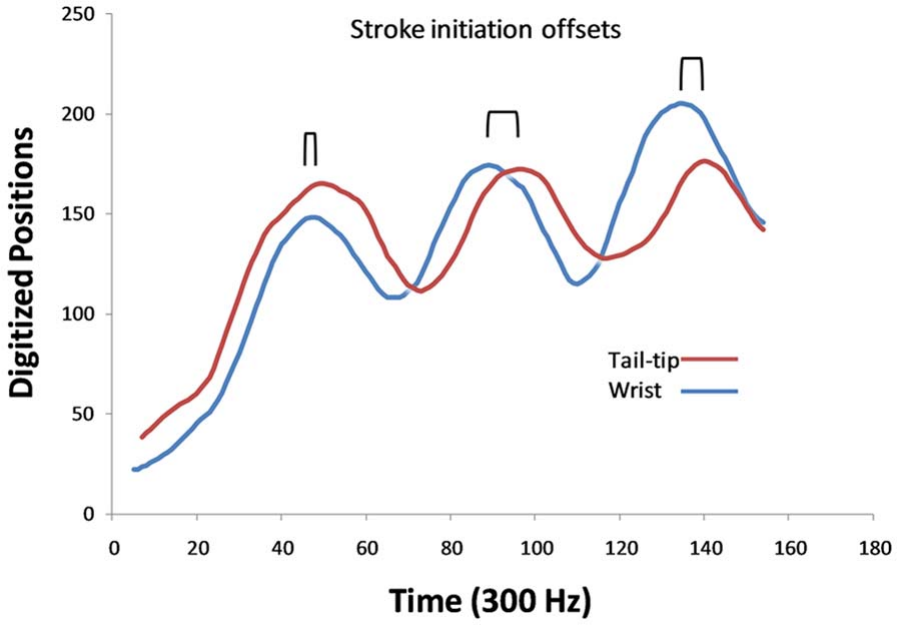
Stroke initiation offsets. Stroke offsets between the left wing and tail-membrane are illustrated by plotting the digitized motions of the left wrist (blue line) and tip of the tail (red line) for the little brown myotis (*Myotis lucifugus*) through three wingbeat cycles. In this example, the individual used intitiation offsets of the upstroke and downstroke timings in order to produce asynchronous flapping between the wings and the tail-membrane. Black lines indicate degree of divergence in timing of downstroke motions (oscillation offsets).

**Figure 3:**
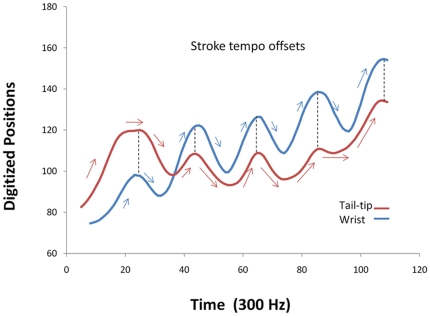
Stroke tempo offsets. Stroke tempo offsets between left wing and tail-membrane as illustrated by plots of the digitized motions of the wrist (blue line) and tail tip (red line) for the fringed myotis (*Myotis thysanodes*) across wingbeat cycles one through five. In this example, the individual showed little to no stroke intiation offsets, but instead used differential flap rates between the wings and tail-membrane to afford asynchrony of motion. The angles of the red and the blue arrows indicate oscillation offsets via stroke tempo.

At slowest cycling speeds, the tail-membrane was flapped at only 72% of the wing cycle speed (wing-minimal-cycle-time = 0.084 seconds, tail-membrane-minimal-cycle-time = 0.117 seconds), whereas during the fastest cycle speeds, the tail-membrane flap was 39% faster than the fastest wing cycling speed (wing-maximal-cycling-speed = 0.054 seconds, tail-membrane-maximal-cycling-speed = 0.033 seconds). Thus, the tail-membrane was employed over a wider range of cycling speeds than observed for the wings and in most cases an individual's kinematic integration of the tail-membrane appeared dampened (reduced fanning motion) as they accelerated during takeoff. As mentioned, relative cycle timings between the wings and tail-membrane did vary among wing-beats within an individual takeoff event. For example, in the little brown bat (*M. lucifugus*)(Figure 2), integration of the tail-membrane and wings varied across the first three wing-beat cycles and in this case, most oscillation offsets occurred in wing-beat cycles two and three. For the fringed myotis (*M. thysanodes*), similar degrees of oscillation offset occurred across all five wing-beat cycles with similar intensity (Figure 3).

There were also two main ways in which bats produced oscillation offsets between wings and the tail-membrane. Most commonly, lagging the downstroke of the tail-membrane relative to the wings was achieved by what we term ‘stroke-initiation-offsets’ wherein the tail-membrane was held at its top position as the bat initiated its wing downstrokes (Figure 2). However, once the tail-membrane downstroke was initiated, the cycle speed was very similar to that of the wings. A second method involved what we term ‘stroke-tempo-offsets’ wherein the initiation of the downstroke of the wings and tail-membrane were synchronized, however, the tail-membrane and wings were cycling at different tempos (Figure 3).

### Performance at takeoff and very slow flight speeds

Wing performance in bats at very slow flight speeds differs from performance at higher flight speeds [18], [19] and analysis of trailing vortices indicate that the lift and thrust generated during the wing's upstroke are minimal at very slow flight speeds (1–3 m ^s−1^) [17]. Although we currently do not have data on the amount of thrust produced by the tail-membrane during horizontal takeoffs at very slow flight speeds (<1 m ^s−1^), the offset oscillations produced by delayed initiation or slower relative downstroke speed and the high level of tail-membrane fanning effort (Video S4) would logically indicate that the tail-membrane is providing an important contribution to takeoff acceleration.

**Figure 4:**
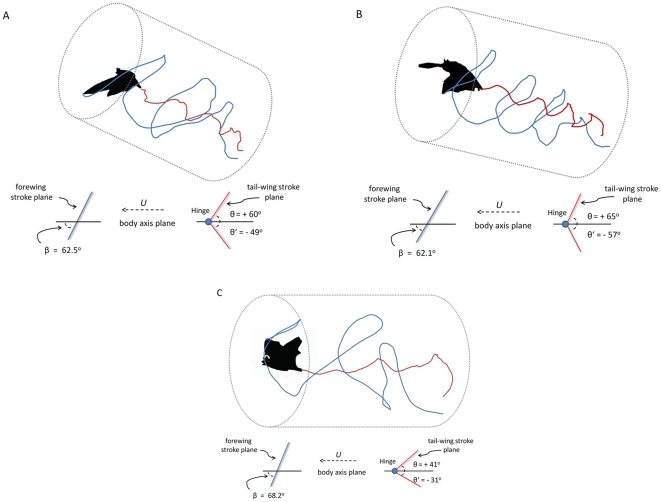
Takeoff trajectory and wing versus tail-membrane motions. Digitized tracings of left wing tip (blue) and tail tip (red) for three takeoff trajectories observed in little brown myotis (*Myotis lucifugus*). Angle of flight trajectories are represented by tilt of each cylinder. Data are provided below each cylinder indicating least degree of left wing stroke angle to body plane (β) and maximal tail-membrane stroke angles above the body plane (θ = +angle) and below the body plane (θ′ = −angle) for each individual adjusted to the horizontal for comparison. Hinge indicates a pivot-point presumably at the sacral/caudal vertebral joint. Body plane is indicated by the dotted arrow and *U* = velocity.

Analysis of digitized tracings (Figure 4) of the left wingtip path and tail-tip path for little brown myotis (*Myotis lucifugus*) showed variation of stroke planes as related to takeoff trajectories. An individual with a relatively steep takeoff trajectory for this species (Figure 4A, 34° to the horizontal) displayed a shallow wing stroke plane relative to the body plane (β = +62.5°) and the tail-membrane stroke plane exhibited a relatively moderate degree of fanning motion (dorsal tail-membrane extension, θ = 60°, ventral tail-membrane flexion, θ′ = −49° with 109° total sweep degrees). An intermediate takeoff angle of 24° to the horizontal (Figure 4B) by another individual produced a wing stroke plane (β) of 62°, and a degree of dorsal tail-membrane extension and ventral flexion angles (θ) of +65.3° and (θ′) of −57° respectively, resulting in 122.5° of total tail-membrane fanning motion. An individual with a takeoff angle horizontal to the platform (Figure 4C, 0°) displayed a wing stroke plane angle (β) of 68° and the least degree of tail-membrane fanning motion (θ = +41° , θ′ = −31°, total tail-membrane sweep was 72°).

**Figure 5:**
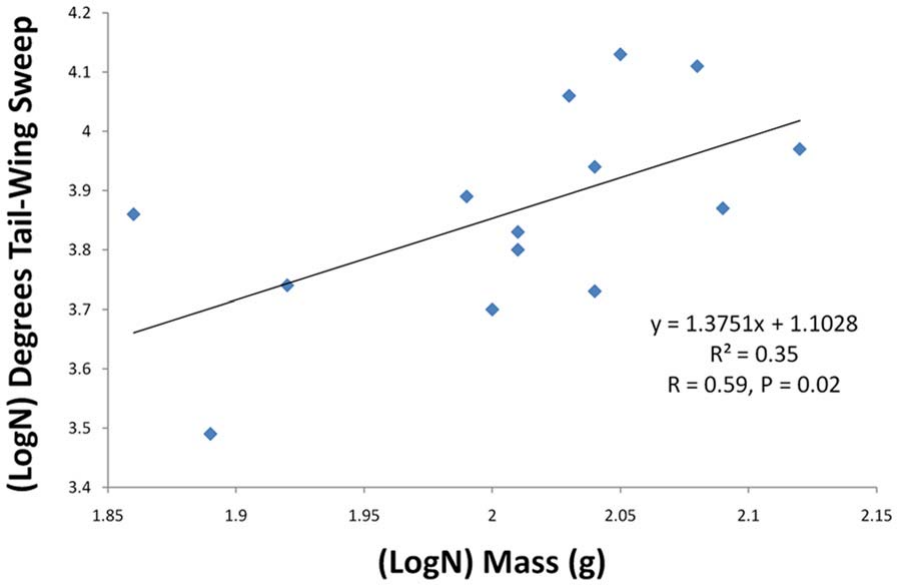
Tail-membrane extension relative to body mass. Model two regression analysis showed a significantly positive relationship between (LogN) dorsal extension of the tail-membrane above the body plane and (LogN) mass (g) of individual little brown myotis (*M. lucifugus*), indicating that degree of dorsal extension of the tail-membrane is mechanically adjusted relative to mass to be lifted.

The average extent of dorsal tail-membrane extension measured as degrees above the horizontal axis of the body in 14 male little brown myotis (*M. lucifugus*) was 48.5° (range 32.7°–49.2°, SD = 8.3°). Extent of dorsal tail-membrane motion above the body-axis as logN-logN plotted against body mass (g) showed a significantly positive correlation (Figure 5, R^2^ = 0.33, R = 0.59, P = 0.02). Total degrees of arc regarding tail-membrane fanning motions in *M. lucifugus* ranged from 72.5° to 134.9° and these data did not significantly correlate with body mass (R^2^ = 0.13, R = 0.368, P = 0.30). Thus it appears that dorsal extension of the tail-membrane is a primary way in which vespertilionid bats increase thrust support during horizontal platform takeoffs.

**Figure 6:**
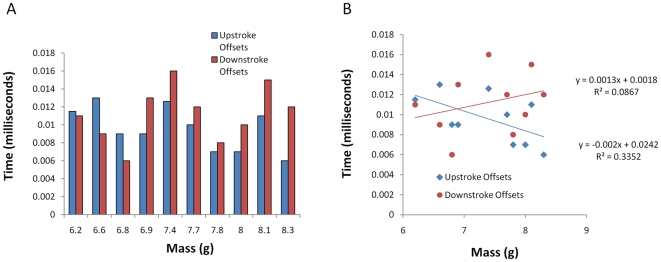
Change in oscillation offsets relative to body mass A. Histogram showing extent of oscillation offsets between the left wing and tail tip during the upstroke (blue bars) and downstroke (red bars) for the little brown myotis (*M. lucifugus*), plotted against individuals that varied in body mass. **B.** Regression plot of the offset data shows the switch-over point of regression lines where downstroke offsets (red line) become more pronounced than upstroke offsets (blue line) in relation to body mass. Although neither regression line is statistically significant due to high variation in the sample, the consistent trend towards greater offsets occuring during downstrokes as body mass increases suggests meaningful shifting of kinematics with mass.

There was also a change in the relationship between upstroke versus downstroke oscillation offsets between wings and tail-membrane motions in relation to body mass in *M. lucifugus* (Figure 6A). At lower body mass, upstroke offsets between the wings and tail-membrane were greater than observed in downstroke offsets. However, when mass was greater than 6.8 g, downstroke offsets became more pronounced than upstroke offsets. Regression analysis (Figure 6B) showed the switchover in offset timings between upstrokes and downstrokes of the wings and the tail-membrane relative to body mass. Although neither regression line is significant due to high variation (upstroke: R^2^ = 0.33, R = 0.57, P = 0.08; downstroke: R^2^ = 0.09, R = 0.29, P = 0.41), the consistent switchover pattern underscores a potential change in kinematics depending on the body mass of an individual, an area of future research.

### Species-specific patterns of tail-membrane employment

**Figure 7:**
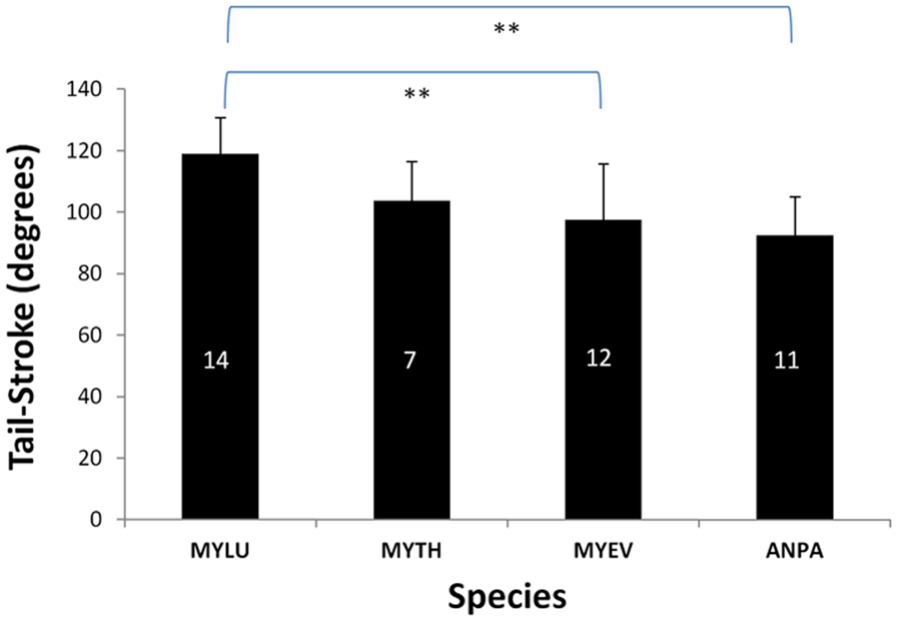
Use of the tail-membrane across species. Integration of ecomorphology with use of the tail-membrane showed significant differences among species (P = 0.01) most broadly separated ecomorpholgically. Mean tail-stroke values (degrees of arc across the body-axis) were highest for the little brown myotis (MYLU) that has the highest wing loading and a preference for open-aerial flight as compared to the other three species. The ground foraging pallid bat (*Antrozous pallidus*, ANPA) has the lowest wing loading of the group and had the shortest tail sweep on average. Post-hoc pair-wise comparisons showed that MYLU was signficantly different from the long-eared myotis (*M. evotis*, MYEV) and ANPA, whereas the fringed myotis (*M. thysanodes*, MYTH) was not significantly different from any other species . ** = significant difference at the P = 0.01. Connecting lines show which groups were signficant different. See Table 1 for details.

We calculated the degrees of tail-stroke fanning sweeps for 44 individuals of the four vespertilionid species with the highest sample sizes and that varied in wing loading, flight ecology, and ecomorphology (Figure 7). The average extent to which the tail-membrane was used during horizontal takeoffs was greatest for *M. lucifugus*, known to be an aerial-pursuit forager in open habitats and having the highest comparative wing loading of the group we tested. The ground foraging pallid bat (*A. pallidus*) has the lowest wing-loading of the group and showed the least use of the tail-membrane during takeoffs. However, because this species hunts large terrestrial prey such as scorpions, ground beetles, and centipedes, the use of the tail-membrane may be prompted more when launching from the ground with a prey item in its mouth large enough to significantly increase wing loading. The two other myotis species analyzed showed intermediate use of the tail-membrane between these two extremes. Post hoc Tukey-Kramer Multiple-Comparison Test (Table 1) denoted significant differences between *M. lucifugus* and the two species with lowest wing loadings (*A. pallidus* and long-eared myotis, *M. evotis*); whereas the fringed myotis (*M. thysanodes*) that has intermediate wing loading and is adapted for aerial-pursuit of insects in cluttered habitats, used its tail-membrane in an intermediate fashion that was not significantly different from either *M. lucifugus* and *M. evotis* (DF = 35, Critical Value = 3.814, Alpha = 0.05, Table 1).

**Table 1:** Post hoc Tukey-Kramer Multiple-Comparison Test showed significant differences (DF = 35, Critical Value = 3.814, Alpha = 0.05) among vespertilionid species in degree of tail-membrane sweep during takeoff from a horizontal platform.

Group	Count	Mean	Significantly Different Groups
**MYLU**	14	118.90	ANPA, MYEV
**MYEV**	12	96.82	MYLU
**MYTH**	7	103.83	NONE
**ANPA**	11	92.39	MYLU

MYLU = *Myotis lucifugus*, MYEV = *M. evotis*, MYTH = *M. thysanodes*, ANPA = *Antrozous pallidus*.

## Discussion

Herein we report on extensive use of the tail-membrane as a potentially important mechanical contributor to flight mechanics in bats during very slow take-off flights from a horizontal platform. The way and the extent to which the tail-membrane in vespertilionid bats is used have not been observed in any other flying vertebrate. We show that the tail-membrane can be incorporated in an extensive, complex, and flexible manner (Video S5) as a tail-wing that can be called upon to produce rearward thrust at slower flight speeds. Offsets in timing by lagging the downstroke of the tail-membrane behind the wings appears to result in rearward thrust during moments of the wing upstroke when minimal lift is being produced [18], [19]. The full importance and adaptive nature of this form of flight will require further investigation such as interactions between wings and tail-membrane vortices, but herein we supply the foundation of adaptive and controlled use of the tail-membrane in vespertilionid bats, the extent to which had not been previously documented or calibrated. Evidence indicates that the tail-membrane is used in well-coordinated flight mechanics and shows plasticity by individuals in its use depending upon immediate needs for flight support. In addition, use of the tail-membrane during horizontal takeoffs matches predictions based on wing-loading and ecomorphology of insectivorous bats. We term this mode of flight, ‘Tail-Assisted-Flight-Thrust’ (TAFT) that is initiated by fanning motions of the tail-membrane in producing rearward thrust.

### Hypothetical model for contributions of the tail-membrane during takeoff flight

**Figure 8:**
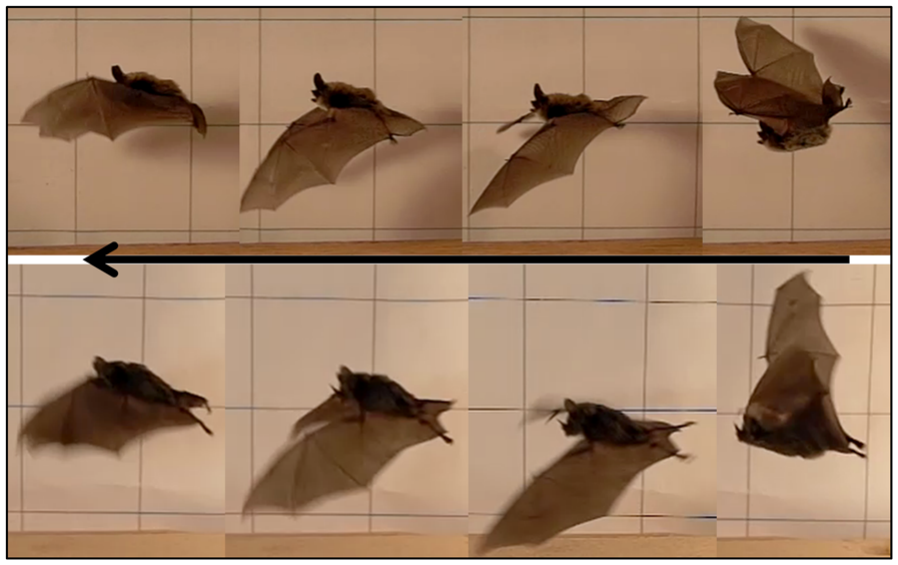
Comparative takeoff dynamics. Using comparable still images between species (upper, long-eared myotis, *Myotis evotis*; lower, short-tailed fruit bat, *Carollia perspicillata*) we show how the long-eared myotis used extensive dorsal extension of tail-membrane to initiate rearward thrust production, whereas the short-tailed fruit bat did not.

Comparative motions of the tail-membrane between the long-eared myotis (Vespertilionidae: *Myotis evotis*) and the short-tailed fruit bat (Phyllostomidae: *Carollia perspicillata*) allowed for insightful comparisons during horizontal takeoffs (Figure 8). We filmed five short-tailed fruit bats multiple times and none showed dorsal extension of the hind-limbs or caudal vertebrae during horizontal launches as was clearly evident in vespertilionid bats. In *C. perspicillata*, there were some lateral and medial motions of the hind-limbs, but this pattern was inconsistent and in some individuals was not evident. There is some evidence using a simplified bat model based on the long-eared bat, *Plecotus auritus* [11], that positioning the tail-membrane during wind tunnel flights at speeds near 6 m ^s-1^ significantly influences flight control. Thus for *C. perspicillata*, leg motions observed during takeoff launches in some individuals may be to adjust the camber of the wings.

Use of the tail-membrane in vespertilionid bats in many ways mimics the fanning motions of the flukes in swimming mammals like dolphins [21], [22], [23], [24] and, in particular, the manatee [20], [25]. Paddle-formed tails in fish and manatees have low aspect-ratios, function around a single hinge (similarly to a rounded, paddle-formed human hand fan), and are efficient in producing forward thrust at low-speeds [17], [25]. The motion of the bat's tail-membrane mimics this fanning motion, however, unlike that observed in aquatic and semi-aquatic animals, the bat's tail-membrane is unfolded only during the downstroke. The fact that bats fold up the tail-membrane during the upstroke phase apparently to reduce negative lift forces and expand it during the downstroke phase to apparently accentuate rearward air displacement agrees with expected airfoil kinematics . In addition, tail-membrane fanning motions in bats are controlled by three pivot points, the hip-joints and the sacro-caudal joint of the tail that must be coordinated, and this differs from the single, central-axis control of the flukes in dolphins and manatees [21], [23], [25].

**Figure 9:**
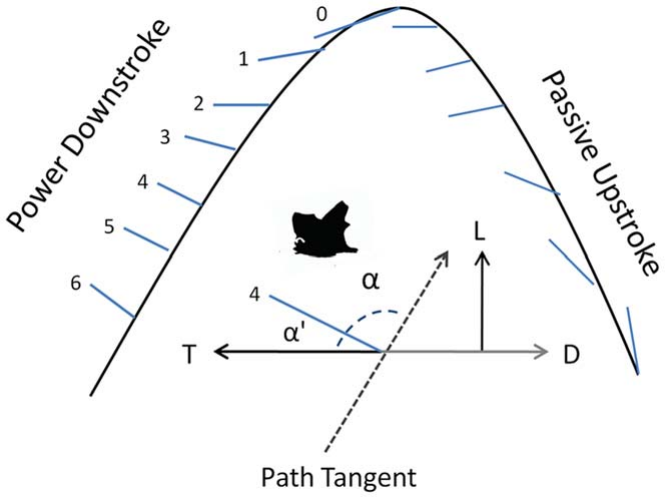
Angle of attack of the tail-membrane during fanning motion. Graphic illustrating how the tail-membrane produces foreward thrust during a platform takeoff. The tip of the tail moves along a sinusoidal path through each stroke cycle represented by the curved black-line. Blue lines indicate the position of the tail-membrane relative to the sinusoidal path. The upstroke of the folded tail-membrane would likely be aerodynamically passive, whereas the downstroke provides a thrust force realtive to pitch angle and angle of attack. Numbers indicate tail-membrane position from top to bottom for a single downstroke. T = Thrust, L = Lift, D = Drag. Inset diagram illustrates how angle of attack and pitch angle were calculated for each tail-membrane position during the downstroke. A path tangent was drawn for each point position of the tail tip. α is the angle of attack of the tail-membrane relative to path (here illustrated for position 4 of the downstroke).

That the tail-membrane displaces air in a direction that would produce rearward thrust is consistent with the physics of fanning motion [17](Figure 9) and it is difficult to conceive of alternative reasons that vespertilionid bats would place so much effort in tail-membrane fanning if the resulting contribution was not significant. Our video of *C. perspicillata* shows that this species typically flapped directly off the horizontal platform rather than performing a push-off as observed in the vespertilionid bats and *C. perspicillata* showed no evidence of attempted tail-mebrane flapping.

In vespertilionid bats, as the tip of the tail moves along a sinusoidal path through each stroke cycle, the position of the tail-membrane changes relative to that sinusoidal path. This why we predict that the downstroke provides a thrust-force similar to that produced by a dolphin's or manatee's tail during slow-speed swimming or even a simple hand-fan when waved for cooling. We calculated both angle of attack (α = angle of tail-membrane axis relative to sinusoidal path) and pitch angle (α′ = angle between tail-membrane axis and translational movement of the animal) through one cycle. Attack angles by position were as follows: 0 (top): 171°, 1: 146°, 2: 126°, 3: 105°, 4: 98°, 5: 94°, 6 (bottom): 90°. Pitch angles by position were as follows: 0: 73°, 1: 60°, 2: 90°, 3: 106°, 4: 119°, 5: 119°, 6: 128°. Greatest thrust production from fanning would hypothetically be produced when the tail-membrane is moved through position 1–4 as pitch angle passes through 90° with an angle of attack of 126°. Unlike the wings, the tail-membrane would predictably perform fanning uniformly along its span, thereby generating much greater propulsive force than lift [17].

Curiously, relative to maximal total degrees of tail-membrane sweep, *M. lucifugus* (134.9° of arc) used about a 40% greater tail-sweep than observed in female bottle-nosed dolphins (54° of arc) [21], [22], [23], [24]. Of course, because of the largely different densities of water versus air, the efficiency of these motions will vary greatly between these two groups.

### Use of the tail by birds during takeoff flight

Use of the tail during flight by birds is also understudied. However, during takeoff in pigeons (*Columba livia*), the majority of tail muscles underwent ‘conspicuous changes in the timing of activity,’ whereas the wing muscles retained the same pattern (mono- or biphasic) of activity in all flight modes (takeoff, slow level flapping flight, and landing) [26]. These data point to a decoupling of neuromuscular control patterns between the wings and tail flight surfaces. Although the tail did not undergo dramatic oscillations during any modes of flight in pigeons, the associated muscles showed changes in activity timing and intensity not exhibited in the standardized temporal activation of the forewing musculature [26]. Pigeons used the tail to minimize drag in order to help themselves accelerate during takeoff in which the tail stroke plane was tilted steeply downward supporting the authors hypothesis that this position would push air rearward to help accelerate the bird forward. The tail stroke plane in pigeons was tilted upward during landing implying that an upward-tilted stroke plane pushes more air forward to slow the bird down [27]. Consequently the tail in birds appears to have a stabilizing role and the use of the caudal musculature during takeoff appears to be used in fixing tail positions that reduced drag [27], as opposed to what we are observing in vepsertilionid bats where tail fanning motions are consistent in generating rearward flight thrust.

### Is the use of the tail membrane in bats novel?

We argue that the use of TAFT locmotion by incorporating fanning of the tail-membrane to produce rearward thrust, thereby acting as a tail-wing in bats, represents a novel form of vertebrate flight that mimics thrust production produced by tail motions in some aquatic organisms. Although some of the hind limb motions we observed in vespertilionid bats may act to adjust camber of the wings, the extent to which they use the tail-membrane far exceeds what would be required for that task. Our comparison of horizontal takeoff between the short-tailed fruit bat (*C. perspicillata*) that has only a partial tail-membrane and our vespertilionid species underscores how extensively the latter uses the tail-membrane for generating rearward thrust during takeoff. In addition, contraction and expansion of the tail-membrane during the stroke cycles clearly conform to predicted power-stroke kinematics.

**Figure 10:**
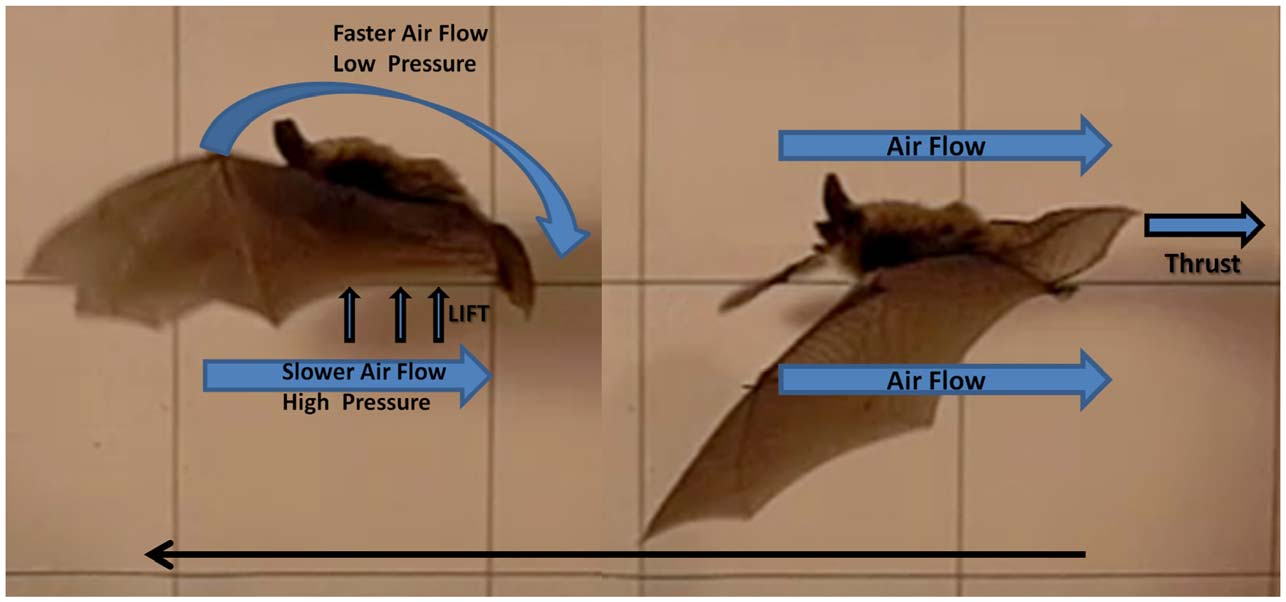
Illustration of hypothetical model of how thrust and lift may be generated. Using the tail-membrane fanning motions (black arrow indicates direction of travel) of a long-eared myotis (*Myotis evotis*), we illustrate a hypothetical duel-use of the tail-wing wherein dorsal extension and downstoke above the body plane delivers thrust, whereas ventral flexion generates thrust and also lift when held in a curved position below the body axis. This positioning would be analogous to an airplane extending its flaps to increase air-speed above a flight surface.

In vespertilionid bats, only the tail-sweep involving dorsal extension of the hind-wing correlated with body mass and thus it appears that dorsal component of the sweep is primarily used to produce rearward thrust. Curiously, all individuals filmed swept the tail-membrane through the ventral flexion component below the body-axis regardless of how much dorsal extension of the tail was used. Thus, it appears that there may be a duel-function of the tail-wing motion relative to its position above and below the body axis. Similar to birds, there appears to be an effort during horizontal takeoffs to either minimize drag when the tail-membrane is moved below the body axis or perhaps bats are using the ventral component of tail-membrane sweep to enhance lift [11] by curving the flight surface to increase wind-speed across the dorsum relative to airflow across the ventrum (Figure 10). This effect would be simiar to an airplane putting down its flaps during takeoff to generate the lift necessary during slow flight speed.

Indeed, we have just begun to uncover the myriad ways in which the tail-membrane may be employed to facilitate navigation of complex terrains and habitats as well as horizontal launches from rock-crevices, buildings, and ground roosts. As is, the tail-membrane kinematics used during platform takeoff is variable among individuals within species, showing high plasiticity in its phasing and integration with the wings. Further, the ease by which individuals use the tail-wing indicates a well-integrated kinematic orchestration among the four limbs, tail vertebrae, and tail-membrane of vespertilionid bats. As with birds [26], there appears be a neurological decoupling of muscle control between the wings and tail-membrane in bats, but this will require EMG data to confirm. That the extensive use of the tail-membrane corroborates aspects of flight morphology such as wing loading and aspect ratio and ecomorpholgical predictions, shows an evolutionary underpinning for use of a tail-wing for thrust and lift production. Future investigations into this phenomenon should include species of bats from other families as well as investigations into the interactions of vortices produced by the wings and tail-membrane and under what ecomorpholgical parameters the tail-wing is employed.

Analysis of the skeletal elements that support fanning motion of the tail-wing will greatly further understanding of the complex nature of tail-membrane adaptations for flight in bats as well as the evolution of flight and species diversity. As with all analyses of flight kinematics, interpretations of, and explanations for potential force production and its effects on flight dynamics requires considerable caution. Herein we put forth our interpretation of how the tail-membrane appears to be used as a supportive tail-wing to produce thrust and lift during takeoff flight from a horizontal platform. Much more sophisticated analyses will be required to test this model.

Lastly, casual observations of high-speed videos provided by other researchers of individuals from other families of bats engaged in skimming water to drink and in nearly vertical flight into caves show clear evidence of TAFT locomotion. Although not yet quantified, we anticipate that most species of bats with full tail membranes, regardless of taxonomic affiliation, use TAFT locomotion and there may be some affiliation with TAFT in the evolutionary origin of flight in mammals.

## Materials and Methods

### Ethics statement

All animal handling protocols were approved by the University of Northern Colorado Institution Animal Care and Use Committee (IACUC protocol number 0505) and field work was carried out under Colorado Division of Wildlife Permit # 11TR897 and a Boulder County Parks and Open Space research permit. All procedures for the handling of bats informed by these permits were followed.

### Animal captures

We used a mist net to capture free-flying bats at a water source located in Boulder County, Colorado. A single mist net was erected in a known flight path. Once individuals were captured and removed, they were placed in cloth sacks to await processing. Each individual was identified to species, sex, reproductive condition and relative age. In addition, for the little brown myotis (*Myotis lucifugus*), we wrapped each individual in a nylon mesh cloth and weighed them to the nearest 0.01 g using a pan-scale (Acculab PP-201, ±0.02; Colombia, MD, USA).

### High-speed videography

We filmed individuals launching from the tailgate of a pickup truck on which a wooden plank was laid for traction. For positional information, we made a back-drop that consisted of 1 m×1 m paper grid divided into 9×10 cm squares. We saturated the filming area using six high-intensity halogen lamps providing 1,800 watts of light. As bats were released, we filmed them from a lateral position to the anticipated flight path using a single high-speed video camera [22], [23], [24] Casio Exilim EX-F1 Digital Camera, (Casio, Inc., Montrose, CA, USA) set at 300 frames per second from a distance of 1 m or 2 m. In some cases, individuals were filmed flying head-on into the camera or from behind, flying away from the camera. These methods were replicated using a wooden plank placed on a table for filming individuals from a captive colony of pallid bats, *Antrozous pallidus*, held at the University of Wyoming, Laramie, USA. The pallid bats were housed in a free-flight room approximately 6×10 m in size and thus were able to maintain their flight skills and abilities.

Bats were not marked because the film resolution was more than adequate to digitize motions of the wing-tip, wrist, and tail-tip throughout the takeoff events without markers and thus minimized potential impacts to wild-caught and released individuals. Markers were placed on landmarks during digitizing analyses through each video frame using analysis software provided by the Tyson Hedrick Research Laboratory at the University of North Carolina, Chapel Hill, USA [28]. Motion angles for the tail-membrane in relation to body plane were calculated using MaxTrax software provided by Innovision Systems (Columbiaville, MI, USA). Body axis was defined and marked as a line between two points, one placed at the nose-tip (as the head is held in-line with the body axis during takeoff flight) and the other placed at the point where tail-membrane connects to the body midline.

Parallax error due to roll and yaw motions of individuals during takeoffs can produce digitizing inaccuracies difficult to correct from a single camera angle. To correct for this, we analyzed film from individuals that showed takeoffs nearing 90 degrees to the camera and had minimal body-axis rotations. If some roll and yaw was evident we made slight adjustment to point placement visually during digitizing by placing markers in positions that allowed for corrections and expected consistency of smooth curvilinear motions of the points through time. We are confident that any parallax errors within our data had minimal, if any, effect on the outcome of our results. We used the body-axis plane of each individual as a reference point to measure sweep angles of the tail-membrane during fanning motions. To produce oscillation graphs for wrist and tail-tip motions in relation to each other as individuals ascended and accelerated from their takeoff points, the motion of the bat's body plane was taken into account, and positional information was adjusted if the body-axis moved when either the wing or tail-membrane were held stationary.

## Supporting Information

Video S1 **Head-on view of a fringed myotis (** ***Myotis thysanodes*** **) using the tail-membrane during takeoff and slow flight.** Note collapsing of the tail-membrane on the upstroke and expansion during the downstroke.
(MPEG)

Video S2 **Rear-view of a fringed myotis (** ***Myotis thysanodes*** **) using the tail-membrane during takeoff and slow flight with extensive use of tail-membrane flapping during seven wing-beat cycles.**
(MPEG)

Video S3 **Rear-view of a Townsend's big-eared bat (** ***Corynorhinus townsendii*** **) during takeoff.** Note the fanning motion of the tail membrane during acceleration and steep climbing.
(MPEG)

Video S4 **Lateral view of a little brown myotis (** ***M. lucifugus*** **) using the tail-membrane during take-off and slow flight.** Although this video was not analyzable due to parallax concerns, it illustrates a view of tail-wing use from various angles.
(MPEG)

Video S5 **Lateral view of a long-eared myotis (** ***Myotis evotis*** **) using the tail-membrane during takeoff and slow flight showing a nearly 90° tail extension during the first upstroke cycle of the hind-wing.**
(MPEG)
